# Case report of streptococcal infection as a potential precipitating factor in cutaneous polyarteritis nodosa in pediatric patients

**DOI:** 10.1002/ccr3.9038

**Published:** 2024-05-31

**Authors:** Pooneh Tabibi, Reza Shiari, Ali Shafiee, Khosro Rahmani, Niloofar Saravi

**Affiliations:** ^1^ Department of Pediatrics Mofid Children's Hospital, Shahid Beheshti University of Medical Sciences Tehran Iran

**Keywords:** atypical presentation, digital ischemia, infectious trigger, migratory arthritis, pediatric vasculitis, polyarteritis nodosa (PAN), streptococcal infection

## Abstract

**Key Clinical Message:**

This pediatric case report underscores the importance of maintaining a high clinical suspicion for polyarteritis nodosa (PAN) in patients presenting with atypical features, such as migratory arthritis and subcutaneous nodules. Importantly, it highlights the focus on the potential relationship between streptococcal infection and cutaneous PAN. Early recognition and prompt, aggressive treatment is critical, as PAN can be a life‐threatening condition if left unmanaged. This case emphasizes the need for a multidisciplinary approach to effectively identify and manage this rare vasculitis disorder in the pediatric population.

**Abstract:**

Polyarteritis nodosa (PAN) is a rare and life‐threatening vasculitis with diverse clinical presentations, posing a diagnostic challenge. Early recognition and prompt intervention are crucial to prevent organ damage. We present the case of an 8‐year‐old boy who exhibited atypical symptoms including migratory arthritis, myalgia, digital discoloration and ischemic changes, and subcutaneous nodules. Initial concerns for septic arthritis were ruled out. A comprehensive evaluation revealed elevated inflammatory markers and a confirmatory skin biopsy demonstrating active leukocytoclastic vasculitis, are highly suggestive of a diagnosis of PAN. Notably, elevated ASO titers suggested a possible concurrent streptococcal infection. The aggressive treatment approach with high‐dose aspirin, steroids, methotrexate, and tocilizumab is justified given the severity of the patient's symptoms and the nature of the disease process. This case underscores the importance of considering PAN in the differential diagnosis for children presenting with atypical features. Early diagnosis and prompt intervention, including addressing potential infectious triggers, are crucial for optimal outcomes in pediatric PAN.

## 1 INTRODUCTION

Polyarteritis nodosa (PAN) is a rare, life‐threatening vasculitis affecting medium‐sized arteries. Its diverse clinical presentations, often mimicking other rheumatologic conditions, pose a significant challenge for timely diagnosis.^1^ This heterogeneity can lead to delays in initiating appropriate treatment, potentially causing irreversible organ damage.^2^ This case report describes an 8‐year‐old boy presenting with atypical features of PAN. The case highlights the crucial role of maintaining a high index of suspicion for PAN, even in atypical presentations. It also focuses on the potential relationship between streptococcal infection and cutaneous PAN, and underscores the importance of a multidisciplinary approach for accurate diagnosis and effective management.

## 2 CASE REPORT

### 2.1 Case history and examination

An 8‐year‐old male presented with a 1‐month history of pain and swelling in his right knee following a minor sprain. The patient, a first‐born child of non‐consanguineous parents, had a birth weight of 3800 grams and current weight of 30 kg. He had a history of normal neurodevelopment and was a native of Afghanistan with no known history of specific diseases. There were no positive points in the family history regarding rheumatologic disorders, and there were no smoker parents. Due to the classic presentation of joint pain and swelling, septic arthritis was suspected. However, knee arthrotomy and joint fluid cultures revealed no bacterial growth. The patient's symptoms progressed to migratory arthritis involving both knees, wrists, and metacarpophalangeal joints over the course of 2 weeks. In addition to the presenting symptoms, he also noticed a blue discoloration of the pulp of his second and fifth fingers on his right hand. Due to worsening symptoms, persistent fever, continuation and worsening of arthritis and joint contracture with severe pain, blue discoloration and concern about progression to necrosis and ischemia, a rheumatology consultation was sought, and the patient was transferred to Mofid Children's Hospital Rheumatology Center in Tehran, Iran.

Upon admission, the patient exhibited a constellation of symptoms including severe illness, pallor, fever, generalized arthralgia with severe pain.

**FIGURE 1:**
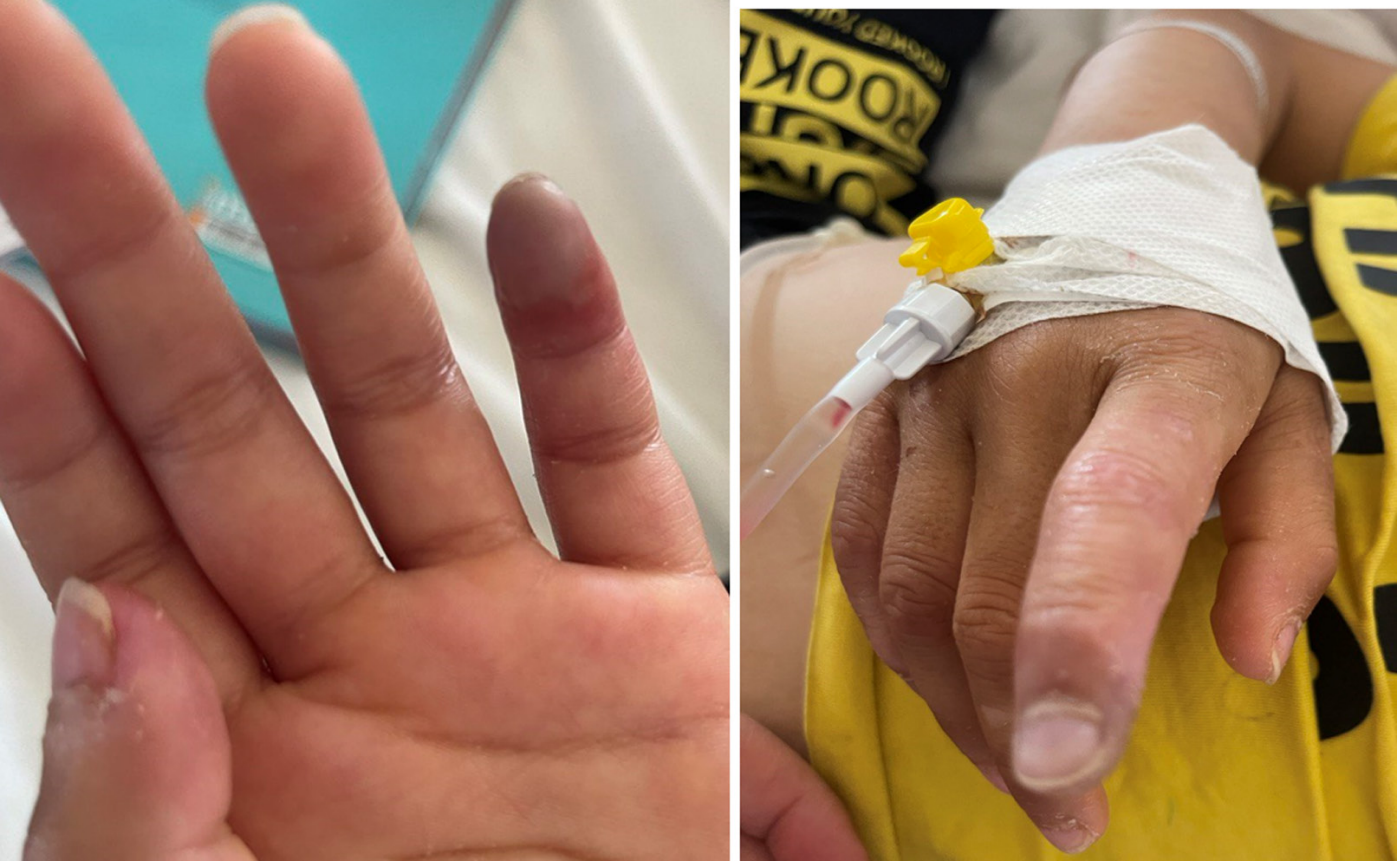
Right hand digital discoloration and infarcts.

On physical examination, the patient exhibited a temperature of 39.5°C, heart rate of 112 bpm, and blood pressure of 110/70 mmHg. He had no Raynaud's phenomenon, livedo, skin ulcers, or ocular lesions. Musculoskeletal examination revealed flexion contractures of both knees, with active arthritis in the metacarpophalangeal and wrist joints. Concerning findings included a blue discoloration and ischemic changes of the pulp of his second and fifth fingers on the right hand, as well as painful subcutaneous nodules on his forearm (Figure 1). The timing of the digital ischemia and color changes did not seem to correlate with cold exposure or winter months, suggesting the vascular involvement was not directly triggered by cold stimuli in this case.

Laboratory investigations revealed elevated inflammatory markers (ESR, CRP, WBC) and abnormal Ferritin level. The hematological, biochemical and immunological findings of the patient are provided in (Table 1). Bone marrow aspiration and thoracoabdomenopelvic CT scan ruled out malignancy.

**TABLE 1:** Hematological, biochemical, and immunological findings of the patient.

Variable	Value	Normal range	Variable	Normal range	Value
WBC (Cell/mm^3^)	**25,500**	4000–12,000	Anti‐CCP	Negative	Negative<10
Neutrophil (cell/mm^3^)	20,400	1500–8500	LDH	718	<746
Lymphocyte (cell/mm^3^)	5100	3000–9500	CPK	48	24–195
Hb (gr/dL)	**7**	11.5–13.5	RETIC	1.5%	0.2–2
MCV (fL)	69.4	77–95	Uric acid (mg/dL)	3.5	3–7
PLT (*10^3^/L)	507,000	150,000–450,000	CRP (mg/L)	**57**	NL <6
ESR (mm/h)	**95**	NL <20	Wright	Negative	Negative<1/80
ANCA/IFA	<1/20	Negative titer <1/20	Coombs Wright	Negative	Negative<1/80
ANA	Negative	Negative<1.40	2ME	Negative	Negative<1/40
RF	Negative	Negative<10	IGRA	Negative	
Ferritin (ng/mL)	291	1.5–205	ASO Titer (IU/mL)	**2850**	Negative<150
C3 (mg/dL)	236	90–180	Interleukin 6	**462.27**	Up to 17
D‐dimer (g/mL)	**800**	Negative<200	HIV	Nonreactive	
Fibrinogen (mg/dL)	403	200–400	HBs Ag	Nonreactive	
UREA	14	12–45	HCV‐Ab	Nonreactive	
Creatinine	0.5	0.2–1.4	PT	12.5	11–13.5
AST (IU/L)	**78**	0–35	PTT	27	25–35
ALT (IU/L)	**70**	0–45	INR	1	0.8–1.1
Urine analysis	Normal		Blood culture	Neg	

*Note*: As mentioned in above table, as a bold values; our patient has, leukocytosis (elevated white blood cell count), thrombocytosis (elevated platelet count), anemia (low red blood cell count), elevated acute phase reactants.

Abbreviations: ALT, alanine aminotransferase; ANA, anti‐nuclear antibody; Anti CCP, anti cyclic citrullinated peptide; AST, aspartate aminotransferase; CRP, C‐reactive protein; ESR, erythrocyte sedimentation rate; Hb, hemoglobin; INR, International Normalized Ratio; PLT, platelet; PT, prothrombin time; PTT, partial thromboplastin time; RF, rheumatoid factor; WBC, white blood cell.

### 2.2 Treatment, outcome, and follow‐up

**FIGURE 2:**
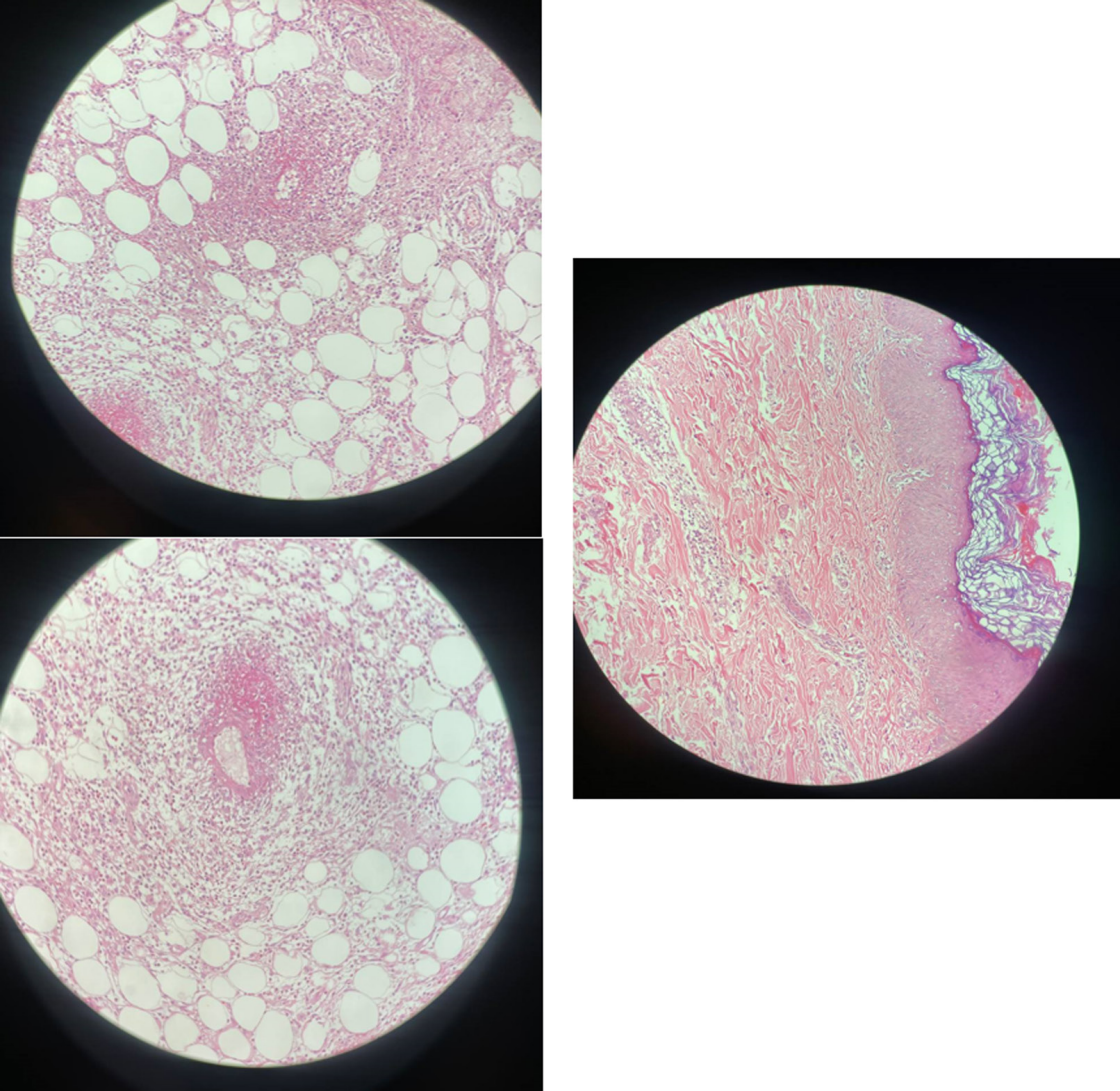
Skin biopsy: histopathological findings. The key features consistent with a vasculitis process include: prominent inflammatory cell infiltrate within the vessel walls and surrounding tissue, composed primarily of neutrophils and eosinophils. Clear disruption and fibrinoid necrosis of the vessel wall architecture, indicating vessel wall damage. Extravasation of red blood cells into the perivascular tissues, suggestive of hemorrhage. These histopathological changes correlate well with the clinical description in the case report of a “painful, erythematous skin rash, subcutaneous nodules” on the patient's forearm. The pathology findings directly support the vasculitis nature of the cutaneous manifestations in this polyarteritis nodosa case.

Despite initial broad‐spectrum antibiotic treatment, the patient continued to experience persistent fever and elevated inflammatory markers. Interestingly, laboratory tests showed elevated IL‐6 and ASO titer, suggesting a possible infectious trigger. Based on these findings, a high‐dose regimen of acetylsalicylic acid (ASA) (50 mg/kg/day QID for 2 weeks, tapered) and a single dose of penicillin G benzathine (IM) with subsequent prophylaxis every 3 weeks led to a dramatic improvement in fever, arthritis, and arthralgia. However, a skin biopsy confirmed the diagnosis of PAN, revealing active leukocytoclastic vasculitis with fibrinoid necrosis of medium‐sized deep dermal and hypodermal vessels (Figure 2).

The presence of severe illness, high fever, generalized arthralgia, and the progression to involve multiple joints (both knees, wrists, and metacarpophalangeal joints) point to a systemic vasculitis process, Therefore, despite the lack of radiographic evidence of aneurysms or stenosis on CT angiography, and the absence of overt renal, cardiac, or other organ involvement, the overall clinical presentation, laboratory findings, along with the skin biopsy findings of leukocytoclastic vasculitis and fibrinoid necrosis, are highly suggestive of a diagnosis of cutaneous PAN (CPAN). The aggressive treatment approach with high‐dose aspirin, steroids, methotrexate, and tocilizumab is justified given the severity of the patient's symptoms and the nature of the disease process. Oral prednisolone (2 mg/kg/day) was initiated with gradual tapering. Additionally, to minimize corticosteroid side effects, methotrexate (10 mg/m^2^) was introduced as a disease‐modifying antirheumatic drug (DMARD) for long‐term management. Furthermore, Tocilizumab, an interleukin‐6 receptor antagonist, was added to target the potential IL‐6 pathway in the inflammatory process. Finally, Enoxaparin, an anticoagulant, was administered to prevent blood clot formation in the affected vessels. Fortunately, the implemented treatment regimen led to a remarkable improvement in the patient's condition. Notably the initial discoloration, threatened necrosis of the fingers and subcutaneous nodules, resolved completely. Additionally, the patient experienced a significant reduction in fever episodes and a marked improvement in joint pain and arthritis with overall well‐being. To assess for potential cardiac involvement, an echocardiogram was performed, revealing normal findings. Subsequent monthly follow‐up with echocardiography and laboratory tests continued to show positive results, with no evidence of cardiac involvement. These positive outcomes highlight the effectiveness of the combined therapeutic approach.

## 3 DISCUSSION

PAN is an autoimmune disorder characterized by necrotizing inflammation of medium‐sized arteries, leading to tissue ischemia and potential multi‐organ involvement.^3^ While the classic presentation of PAN in adults includes fever, arthralgias, peripheral neuropathy, myalgia, and visceral symptoms, children with PAN often exhibit a more heterogeneous clinical picture, significantly hindering timely diagnosis.^2^, ^4^ This heterogeneity, coupled with the relative rarity of PAN in the pediatric population, creates a substantial diagnostic challenge.^2^, ^4^ Cutaneous PAN (CPAN), a variant characterized by typical skin lesions and limited systemic symptoms, presents an even greater challenge due to its even lower prevalence compared to classical PAN.^2^, ^3^ The exact cause of CPAN remains elusive, although theories exist suggesting a potential role for viral infections or other environmental triggers in genetically predisposed individuals.^4^, ^5^ As exemplified by our case, the diverse clinical manifestations of PAN contribute to its diagnostic complexity.^3^ Our patient presented atypically with migratory arthritis, digital ischemia, and subcutaneous nodules, leading to an initial, alternative working diagnosis. This deviates from the more common presentations in children, which often include abdominal pain, livedo reticularis, and constitutional symptoms like fever and malaise.^4^, ^6^ Our case highlights the difficulty of pinpointing PAN in atypical presentations, as evidenced by the initial suspicion of septic arthritis due to the joint pain and arthritis. Fortunately, negative joint fluid cultures, elevated inflammatory markers, and ultimately, a confirmatory skin biopsy, steered us towards the correct diagnosis of PAN.^7^ Compared to other reported pediatric PAN cases, our patient exhibited a unique combination of clinical features, including the migratory arthritis and digital ischemia, which are less commonly reported in the literature.^5^, ^6^, ^8^ This case underscores the importance of maintaining a high index of suspicion for PAN in children, even when presenting with atypical features. A multidisciplinary approach involving rheumatologists, infectious disease specialists, and potentially dermatologists is crucial for accurate diagnosis and management of PAN in children. Collaboration among these specialists allows for a comprehensive evaluation, considering differential diagnoses and implementing appropriate diagnostic tests.^9^, ^10^

Differential diagnoses considered in our case included:

1. Septic arthritis: Ruled out by negative joint fluid cultures.
2. Juvenile idiopathic arthritis (JIA): While JIA can cause arthritis and skin lesions, the migratory pattern, finger ischemia, and elevated inflammatory markers were more suggestive of PAN.

Malignancy disorders: ruled out by normal BMA and CT scan.

Early diagnosis and prompt initiation of treatment are critical to prevent irreversible organ damage in PAN and improve long‐term prognosis in children.^11^ The standard treatment approach for PAN typically involves a combination of immunosuppressive therapy, glucocorticoids, and supportive measures.^9^, ^11^

1. Immunosuppressive therapy: (such as cyclophosphamide or methotrexate) to suppress the immune response and induce remission.
2. Glucocorticoids: (like corticosteroids) to control inflammation.
3. Supportive measures: including pain management and treatment of any organ‐specific complications.^7^, ^10^, ^11^

Having established the diagnosis of PAN, we initiated treatment with a combination therapy. This included Methotrexate, a Disease‐Modifying Anti‐Rheumatic Drug (DMARD), administered at a dosage of 10 mg/m^2^, alongside corticosteroids like prednisolone 2 mg/kg/day, which effectively controlled the inflammation. This case sheds light on two emerging aspects in PAN management: targeted therapies and the potential role of underlying infections. The use of Tocilizumab, an interleukin‐6 receptor antagonist, in our case management aligns with growing research suggesting the potential benefit of targeting specific inflammatory pathways in severe PAN cases.^12^, ^13^ Additionally, the patient's elevated IL‐6 levels and the initial treatment with penicillin for a suspected streptococcal infection raise the intriguing possibility of an infectious trigger in this case. Several studies have shown a potential association between certain infections and the development or exacerbation of vasculitis.^1^, ^5^ Further investigation into this association could lead to improved diagnostic and treatment strategies.

While the presenting symptoms were predominantly cutaneous, the presence of systemic features such as fever and elevated inflammatory markers suggests possible systemic involvement. However, importantly, there was no evidence of internal organ involvement based on radiographic findings (e.g., no aneurysms or stenosis on CT angiography) and no overt renal, cardiac, or other organ dysfunction. This suggests the vasculitis process was largely confined to the skin in this case. It's noteworthy that despite the initial presentation of arthritis and fever, and the need for aggressive treatment, the patient's course ultimately responded well to a limited treatment regimen of low‐dose corticosteroids and methotrexate, achieving complete remission on medication. This limited treatment course further supports the possibility of a primarily cutaneous vasculitis. Additionally, the correlation of the high ASO titer as well as the presence of subcutaneous nodules in this case is highly suggestive of a streptococcal infection‐associated etiology, similar to the presentation of acute rheumatic fever (ARF) with cutaneous manifestations. This connection to a preceding streptococcal infection further supports the diagnosis of cutaneous PAN in this patient.^5^

Together, the lack of systemic organ involvement, the favorable response to limited immunosuppressive therapy, and the association with streptococcal infection all point towards a predominantly cutaneous vasculitis process in this case of PAN.

We also believe there are several potential contributing factors to the patient's anemia; as well as blood loss due to multiple joint arthrotomy procedures, these invasive procedures can result in blood loss, which can exacerbate or precipitate anemia in this setting. The underlying PAN has led to a state of chronic inflammation in the patient. This chronic inflammatory state can contribute to the development of anemia through several mechanisms. In addition to the chronic inflammation associated with PAN, Streptococcal infections can also trigger a chronic inflammatory response, further exacerbating the anemia of chronic inflammation.^1^, ^5^

Our case highlights several additional important points. First, PAN can present atypically even in pediatric patients. Second, a high degree of suspicion and a multidisciplinary approach involving rheumatologists, dermatologists, and other specialists are crucial for accurate diagnosis. Third, identifying and treating any underlying infections, such as the streptococcal infection in this case, can significantly improve the patient's outcome.^7^, ^11^ In comparison to other reported pediatric PAN cases, our management approach involving the use of targeted biologics, such as tocilizumab, and the consideration of an underlying infectious trigger, represents a more comprehensive and personalized approach to disease management.^13^ This case highlights the importance of an individualized treatment plan, taking into account the patient's unique clinical presentation and potential contributing factors.

Our case report provides valuable insights into the presentation and management of a patient with PAN. The detailed documentation of the unique clinical features, progression, clinicopathological correlations, and treatment response contributes to our understanding of the heterogeneous nature of PAN and cutaneous PAN (CPAN). However, it is important to acknowledge several limitations of this report. Firstly, cryoglobulin testing was unavailable, which could have provided further information regarding potential differential diagnoses, such as cryoglobulinemic vasculitis.^14^ Additionally, as this is a single case study, the generalizability of the findings is limited, and further research with larger patient cohorts is warranted. Another limitation is the lack of direct immunofluorescence (DIF) testing on the skin biopsy. DIF can be a valuable tool in the diagnostic workup of vasculitides, and its inclusion could have offered additional clinicopathological insights.^15^, ^16^ Furthermore, imaging of the ischemic fingers using contrast‐enhanced MRI or MRA was not performed. Such advanced imaging techniques could have yielded more detailed information about the extent and nature of the vascular pathology involved.^17^ Despite these limitations, the present case report contributes to the broader understanding of PAN and CPAN presentations. The detailed documentation of the clinical features, their progression, and the treatment response provides valuable insights that may guide future management of these complex vasculitides.

## 4 CONCLUSION

This case report highlights the critical importance of maintaining a high index of suspicion for rare but severe conditions like PAN, even when the presenting symptoms are atypical. The association between PAN and preceding streptococcal infection, as observed in this patient, underscores the need for clinicians to carefully consider this potential trigger when evaluating vasculitis disorders. Early recognition of the correlation between streptococcal infection and the development of PAN, along with prompt implementation of a comprehensive, multimodal treatment approach, are essential for optimizing patient outcomes. This case demonstrates that clinicians must be vigilant in considering PAN within the differential diagnosis when evaluating patients with signs and symptoms suggestive of vasculitis or multisystem inflammatory disease, especially in the context of a recent streptococcal infection. Timely diagnosis and aggressive management, as demonstrated in this patient, can be life‐saving. Continued research to enhance our understanding of PAN pathogenesis, including the role of preceding streptococcal infection, is warranted. By remaining cognizant of rare disease presentations and advancing the scientific understanding of PAN, healthcare providers can work to improve the prognosis for this severe condition.

## AUTHOR CONTRIBUTIONS

**Pooneh Tabibi:** Conceptualization; data curation; writing – original draft; writing – review and editing. **Reza Shiari:** Project administration; supervision. **Ali Shafiee:** Data curation; investigation; writing – review and editing. **Khosro Rahmani:** Supervision. **Niloofar Saravi:** Resources; validation.

## FUNDING INFORMATION

None.

## CONFLICT OF INTEREST STATEMENT

The authors declare no competing interests.

## CONSENT

Written informed consent was obtained from the patient to publish this report in accordance with the journal's patient consent policy.

## CONSENT FOR PUBLICATION

The consent for publication was obtained from the patient's parents.

## Data Availability

All data and materials used in this research are available upon request. Researchers interested in accessing the data and materials may contact shiareza@yahoo.com.
